# Expression of Calbindin, a Marker of Gamma-Aminobutyric Acid Neurons, Is Reduced in the Amygdala of Oestrogen Receptor β-Deficient Female Mice

**DOI:** 10.3390/jcm11071760

**Published:** 2022-03-22

**Authors:** Daniel Kalinowski, Krystyna Bogus-Nowakowska, Anna Kozłowska, Maciej Równiak

**Affiliations:** 1 Department of Animal Anatomy and Physiology, Faculty of Biology and Biotechnology, University of Warmia and Mazury in Olsztyn, 10-727 Olsztyn, Poland; boguska@uwm.edu.pl (K.B.-N.); mrowniak@uwm.edu.pl (M.R.); 2 Department of Human Physiology and Pathophysiology, School of Medicine, University of Warmia and Mazury in Olsztyn, 10-082 Olsztyn, Poland; kozlowska.anna@uwm.edu.pl

**Keywords:** anxiety disorders, oestrogen receptor β knock-out mice, amygdala, neuronal loss, GABA impairment, immunohistochemistry

## Abstract

Oestrogen receptor β (ERβ) knock-out female mice display increased anxiety and decreased threshold for synaptic plasticity induction in the basolateral amygdala. This may suggest that the γ-aminobutyric acid (GABA) inhibitory system is altered. Therefore, the immunoreactivity of main GABAergic markers—i.e., calbindin, parvalbumin, calretinin, somatostatin, α1 subunit-containing GABA_A_ receptor and vesicular GABA transporter—were compared in the six subregions (LA, BL, BM, ME, CE and CO) of the amygdala of adult female wild-type and ERβ knock-out mice using immunohistochemistry and quantitative methods. The influence of ERβ knock-out on neuronal loss and glia was also elucidated using pan-neuronal and astrocyte markers. The results show severe neuronal deficits in all main amygdala regions in ERβ knock-out mice accompanied by astroglia overexpression only in the medial, basomedial and cortical nuclei and a decrease in calbindin-expressing neurons (CB+) in the amygdala in ERβ knock-out mice compared with controls, while other markers of the GABAergic system remain unchanged. Concluding, the lack of ERβ led to failure in the structural integrity of the CB+ subpopulation, reducing interneuron firing and resulting in a disinhibitory effect over pyramidal function. This fear-promoting excitatory/inhibitory alteration may lead to the increased anxiety observed in these mice. The impact of neuronal deficits and astroglia overexpression on the amygdala functions remains unknown.

## 1. Introduction

Anxiety disorders are characterised by persistent, overwhelming anxiety and fear, and they affect ~12% of people in a given year and between 5% and 30% over a lifetime [1]. The prevalence of these disorders in women is approximately twice as frequent as in men [2,3]. It has been estimated that in 2017 an estimated 284 million people experienced an anxiety disorder globally, making it the most prevalent mental health disorder [3,4,5,6]. Around 63% (179 million) were females, relative to 105 million males [5,7]. Since anxiety disorders affect so many people [8] and often coexist with other psychiatric conditions such as depression, drug addiction and/or personality disorder [3], they constitute a serious social and clinical problem.

The amygdala is essential for fear and anxiety processing [9], and its function is disrupted in anxiety disorders [10]. Moreover, there is also a strong link between altered amygdala processing and depression [11], personality disorder [12] and/or substance abuse [13]. Interestingly, all these emotional disorders occur with different incidence/severity in men vs. women. For example, anxiety disorder and depression are quoted more often in women [14,15]. In contrast, drug addiction and attention deficit hyperactivity disorder are more common in men [14]. Unfortunately, mechanisms which increase the risk of these diseases in a particular gender are as yet almost unknown.

One of the major factors that may account for sex differences in the incidence/severity of various emotional disorders is altered oestrogen signalling [16,17]. Indeed, a strong association exists between oestrogen and emotional abnormalities in women; e.g., mood swings, anxiety and depression were often linked with low oestrogen levels in women after menopause [18,19]. Oestrogen replacement therapy improves these conditions [20]. Moreover, many reports indicate that the risk of anxiety and depression is greater for perimenopausal women than for premenopausal or postmenopausal women, even after considering various factors such as lifestyle, age, race etc. [21,22]. In addition, behavioural studies in rodents revealed that decreased oestrogen signalling due to oestrogen receptor β (ERβ) knock-out leads to increased anxiety [23,24], while ERβ agonists usually produce strong anxiolytic effects [25,26]. However, little is known about the mechanisms or sites of actions of oestrogen and neurotransmitter systems involved in these complex modulatory processes. There is currently only evidence that in the adult ERβ knock-out mice there are severe cellular deficits in some brain regions, including the medial amygdala, which increase with age [27]. Moreover, these deficits are accompanied by a proliferation of astroglial cells in the limbic structures [27]. There is also evidence reporting a decreased threshold for synaptic plasticity induction in the basolateral amygdala [24], which may suggest that the activity of γ-aminobutyric acid (GABA) inhibitory system is impaired. Interestingly, pharmacological manipulation of GABA type A (GABA_A_) receptors in the amygdala of wild-type mice may imitate the effects of ERβ knock-out [24]. In fact, decreased GABA signalling in the amygdala might be the beginning of alterations in synaptic plasticity in this region, which result in an anxiety increase in ERβ knock-out female mice. Although the expressions of GABA_A_ receptors and glutamate decarboxylase (GAD) were reported to be unchanged in ERβ knock-out mice [24], there are many other possible mechanisms for reducing the inhibitory tone in the amygdala, despite sufficient GABA_A_ and GAD content [28,29]. One of the possible mechanisms may be, e.g., through serotonergic signalling [30,31] and the 5-hydroxytryptamine 1a receptors [32,33], as the number of these receptors is upregulated in ERβ knock-out mice [24].

The amygdala is populated by a large number of γ-aminobutyric acid (GABA)ergic neurons [34], and a large majority of them co-express different calcium-binding proteins and/or neuropeptides [35,36]. These proteins and neuropeptides differentiate GABAergic neurons into discrete subpopulations performing different roles in the inhibitory mechanism of the amygdala. Calbindin (CB), parvalbumin (PV) and calretinin (CR) are usually used to mark these subpopulations in the amygdala [37,38]. In the rat [38] and monkey [39] amygdala, they constitute the bulk of GABAergic neurons. Moreover, axons of parvalbumin-expressing neurons (PV+) target the soma of neighbouring pyramidal cells and cause inhibition [40,41,42], while axons of neurons utilising CB (CB+) primarily innervate spines and distal dendrites of pyramidal neurons [43]. In contrast, CR-containing neurons (CR+) often target other GABAergic interneurons, as they do in the hippocampus and several cortical regions [44,45], and by inhibiting them disinhibit pyramidal neurons [46,47]. It is worth noting that some CB+ neurons and many PV+ cells are endowed with ERβ in wild-type animals, whereas CR+ cells usually contain oestrogen receptors alpha [48,49]. Considering the above, it is very easy to imagine that alterations in the activity of CB+, PV+ and CR+ subpopulations may lead to an alteration in excitatory/inhibitory tone. This may result, for example, in the increased anxiety observed in ERβ mutant female mice.

Thus, the aim of the present study was to investigate, for the first time in the amygdala of ERβ knock-out female mice, the presence/absence of quantitative alterations among the main markers of the GABAergic system. Such alterations could be the natural basis for inhibitory/excitatory imbalance and increased anxiety in these animals. We chose ERβ^−/−^ females rather than ERβ^−/−^ males, as ERβ^−/−^ females are the best validated animal model of reduced oestrogen signalling and anxiety disorders based on genetic, behavioural and neurobiological studies [25,50,51]. The following markers were tested: CB, PV and CR, since the cells expressing these proteins constitute the main subsets of the GABAergic population. Additionally, somatostatin (SOM) was also included in the study because it appeared that during the present investigation only the expression of CB+ neurons was reduced in ERβ knock-out mice while PV+ and CR+ cells were not affected. Since CB+ neurons in the amygdala include two subsets which are separated and do not overlap—i.e., CB+/PV+ and CB+/SOM+ cells [52]—an additional analysis was performed to clarify whether SOM+ cells are affected. Moreover, vesicular GABA transporter (VGAT) and GABA type A receptor with α1 subunit (GABA_A_α1) receptors were also tested since alterations in the content of both these proteins also have a huge influence on GABAergic inhibition. VGAT was never investigated in ERβ knock-out mice, although GABA type A receptor was tested, but using a quite different method [24]. Finally, the immunoreactivity of neuron-specific nuclear protein (NeuN, pan-neuronal marker) and glial fibrillary acidic protein (GFAP, astrocyte marker) was also investigated in the present study. Severe neuronal deficits and astrocyte overexpression were previously reported in the medial nucleus of the amygdala of the ERβ knock-out mice [27], and both of these factors may have an indirect influence on emotional processing [51]. However, the medial nucleus is not strictly linked with anxiety processing [9], whereas the other and relevant amygdala regions were never examined.

## 2. Materials and Methods

### 2.1. Animals

Adult female mice—i.e., ERβ^−/−^ (homozygous B6.129P2-Esr2^tm1Unc^/J, also known as ERβ KO, *n* = 6, aged 6–8 weeks) and ERβ^+/+^ (C57BL/6J, also known as B6, *n* = 6, aged 6–8 weeks)—were acquired from the Jackson Laboratory (Bar Harbor, ME, USA) and transported to the animal house at the Faculty of Veterinary Medicine of the University of Warmia and Mazury (Olsztyn, Poland). Both ERβ^−/−^ and ERβ^+/+^ mice were acclimatised one week and housed in a temperature-controlled (21 ± 1 °C) and ventilated (12–20 air exchanges/h) animal room which maintained a 12/12 h light/dark cycle (lights on from 6:00 to 18:00). They were kept separately in sanitised polypropylene cages in groups of two/three to avoid stress from isolation and had free access to a standard mice grain mixture (devoid of phytoestrogens; LabDiet^®^ JL Rat and Mouse/Auto 6F 5K52) and tap water ad libitum. Animal care and handling were in strict accordance with the European Union Directive for animal experiments (2010/63/EU) and the 3Rs principle (Replacement, Reduction and Refinement); thus, all animals used were registered, and the staff was adequately educated and trained (Certificate No 1267/2015). According to the Act of 15 January 2015 on the protection of animals used for scientific and educational purpose (adopted in Poland), if the only activity is killing an animal for collecting tissues or organs, it is not considered a procedure, so ethics committee approval is not required. All efforts were made to minimize animal suffering and to use the minimum number of animals necessary to generate reliable scientific data.

### 2.2. Tissue Preparation

Following a two-week habituation phase, all ERβ^−/−^ and ERβ^+/+^ mice were subjected to the same laboratory procedures. First, the oestrous cycle was monitored, and the stage of the oestrous cycle was determined directly from vaginal smears. Cycle monitoring is necessary as both ERβ^−/−^ and ERβ^+/+^ show normative patterns of steroid hormone levels [52], and these steroids fluctuate during the oestrous cycle, which may affect the anxiety behaviour, as was described by Dombret [53]. Therefore, to avoid the oestrogen-induced anxiolytic effect, all mice were anaesthetised in metestrus, as this phase is characterized by a low oestrogen level [54]. The mice were then deeply anaesthetised with an intraperitoneal injection of pentobarbital (Morbital, Biowet, Poland; 2 mL/kg) according to the guidelines of the Humane Society Veterinary Medical Association, and after cessation of breathing, immediately perfused transcardially with saline (0.9%) followed by 4% paraformaldehyde (PFA; pH 7.4; 1040051000, Merck, Germany) in phosphate-buffered saline (PBS; P5493, Sigma-Aldrich, Darmstadt, Germany). After perfusion, the brains were dissected and post-fixed by immersion in 4% PFA overnight, washed three times in 0.1 M phosphate buffer (pH = 7.4, 4 °C) and then cryoprotected for 3–5 days in graded solutions (10%, 20% and 30%) of sucrose (363-117720907, ALCHEM, Poland) in 1×PBS at 4 °C. Finally, the brains were frozen and then sectioned in the coronal plane at a thickness of 10 μm with the use of a cryostat (HM525 Zeiss, Germany). The sections were mounted on object slides and stored at −80 °C until further processing.

### 2.3. Immunohistochemistry

Selected amygdala sections from ERβ^−/−^ and ERβ^+/+^ mice were stained using two immunohistochemical methods: immunoperoxidase labelling with 3.3-diaminobenzidine (DAB) as a substrate-chromogen and immunofluorescence. All staining steps were performed at room temperature in humid chambers (Immuno Slide Staining Trays, R64001-E, Pyramid Innovation Ltd., Polegate, UK).

**DAB staining.** To visualize the location and borders of the individual amygdala regions with the use of NeuN (pan-neuronal marker) and populations of neurons expressing CB, PV or CR in these regions, selected brain sections were labelled using the DAB technique (Table 1) described in detail in the authors’ previous papers [55]. Briefly, these sections, after triple washing in PBS, were pre-incubated for 30 min in 0.3% H_2_O_2_ diluted in 99.85% methanol and then for 60 min with a solution of 10% normal donkey serum (diluted in PBS). The sections were then incubated overnight with a solution of primary antibodies, which were diluted in PBS with 1% normal donkey serum and Triton X-100 (0.3–0.5%). The next day, the sections were triple-washed in PBS, treated with the solution of peroxidase-conjugated secondary antibodies (Imm-PRESS Reagent) for one hour, and finally incubated with a 3% DAB solution (Table 1). In the final step, sections were rinsed with water, dehydrated using a series of graded alcohol dilutions (POCH, Poland), cleaned in xylene and mounted in DPX (DPX Mountain for histology; 44581, Sigma-Aldrich, Germany).

**Immunofluorescence.** To visualize SOM, GABA_A_α1, VGAT and GFAP signal in the amygdala, selected sections in both ERβ^−/−^ and ERβ^+/+^ mice were subjected to routine immunofluorescence, with full details in Równiak’s work [43]. Briefly, the sections were triple-washed with PBS and then incubated for one hour with a blocking buffer composed of 0.1 M PBS, 10% normal donkey serum, 0.1% bovine serum albumin, 0.05% thimerosal and 1% Tween. After this, the sections were triple-washed in PBS and then incubated overnight with a solution of primary antibodies directed to SOM, GABA_A_α1_,_ VGAT and GFAP (Table 1). The antibodies were diluted in a blocking buffer. Finally, the sections were rinsed in PBS, incubated for one hour with the secondary antibodies’ solution (Table 1), mounted with carbonate-buffered glycerol (pH 8.6) and coverslipped.

### 2.4. Controls

The primary antibodies used in the study were carefully chosen. The specificity of the primary antisera used in this study was shown by various researchers using these products in previous immunohistochemical studies [27,56,57,58,59,60]. The rabbit antibody against neuron-specific nuclear protein NeuN (ABN78) was positively validated by the manufacturer using Western blotting in mouse brain extracts, showing bands ~48/42 kDa. Moreover, product descriptions of the rabbit antibodies against calcium-binding proteins, such as anti-calbindin (CB-38), anti-parvalbumin (PV27) and anti-calretinin (7697), demonstrate data with immunoblots carried out using the mouse brain extracts, which show specific bands for these proteins at 28 kDa, 12 kDa and 29 kDa, respectively. These datasheets also demonstrate a lack of any immunostaining in sections taken from the brains of CB, PV or CR knock-out mice by using the mentioned antibodies. Finally, the rabbit antibodies against GABA_A_α1 (ab33299) and VGAT (ab5062P) were successfully validated by Western blots in mouse brain homogenates [61,62]. The specificity of the secondary antibodies was evaluated by omitting or replacing primary antibodies with non-immune sera or PBS. The absence of any immunosignal demonstrated specificity. Finally, to check whether the staining was specific, it was always analysed in the blue (UV), green (488 nm) and red (555 nm) fluorescent channels.

### 2.5. Counts and Measurements

To quantify the density of NeuN+, CB+, PV+, CR+ or SOM+ neurons in the studied amygdala nuclei, the stained sections were examined using an Olympus BX51 microscope (Olympus GmbH, Germany) with a digital camera (CC-12, Soft Imaging System, Münster, Germany) and Cell-F software (Olympus, Hamburg, Germany). The following amygdala nuclei were tested: lateral (LA), basolateral (BL), basomedial (BM), medial (ME), central (CE) and cortical (CO). It is necessary to note that the delineation pattern of various amygdaloid nuclei and nomenclature for the amygdala used in the present study were adopted without modifications from the mouse brain atlas of Paxinos and Franklin [63]. For each nucleus in each animal of both ERβ^−/−^ and ERβ^+/+^ mice, NeuN+, CB+, PV+, CR+ or SOM+ cells were counted on fifteen evenly spaced sections (per antigen) arranged from the rostral (bregma = −1.06) to the caudal extent (bregma = −2.30) of the amygdala [63]. The distance between sections was 70–80 μm. Counting on the single strip was always done with a 40× lens and with the use of 347.6 µm × 260.7 µm regions (test frames). These frames were always distributed to ensure coverage of the full area of the analysed nucleus. The number of test frames per nucleus was diversified: LA: 1–4, BL: 2–6, BM: 2–3, CO: 1–2, ME: 4–6 and CE: 2–3. The counts calculated within the single test frame were averaged, and they had to always be recalculated to value a corresponding to the area of 0.04 mm^2^ or 0.04 mm^3^. The mean values from the test frames covering the particular nucleus on the section were averaged. To calculate neuron density in the particular nucleus in the subject, the results from individual strips were averaged. In the last step, values from the individual nuclei were also averaged in each mice group and saved in the format: mean ± standard deviation (SD).

To evaluate the volume density of GABA_A_α1 and VGAT immunoreactive (IR) elements in the amygdala nuclei, the immunofluorescence-stained sections were analysed according to the automated line scan analysis described and validated by Sathyanesan et al. [64]. Briefly, images acquired with Olympus BX51 microscope were feature-extracted using a Hessian-based filter included in the plugin called FeatureJ [65] for NIH ImageJ software (version 1.53e). These images were then subjected to line scan profile analysis using five lines oriented horizontally (parallel scans) and five lines oriented vertically (perpendicular scans). In the next step, the line scans were baseline-adjusted and processed with the algorithm for peak-detection. Estimation of baseline depends on how peaks (signal) are distinguishable from the background. To evaluate the background value for the line scans, these scans were also always drawn from several immune-negative places. The mean was then calculated from these measurements and constituted the final background value for the single section (threshold). Such a threshold is required for a peak-detection algorithm. Finally, the volume density of GABA_A_α1 and VGAT was calculated using the formula presented by Sathyanesan [64]. The same images were also subjected to signal intensity evaluation using measure analyses by integrated density (IntDen) in NIH ImageJ software. All the measurements in all nuclei in the subjects were determined on ten evenly spaced sections (per antigen) on images covering the whole cross-section area of the nuclei.

The GFAP immunoreactivity was analysed on five evenly spaced sections without cell counting, since the difference in immunoreactive signal was so evident in the selected amygdala regions between ERβ^−/−^ and ERβ^+/+^ mice that cell counts were not necessary.

All measurements were determined by the first author on coded slides, and then the analyses were repeated by two independent researchers, all being blind to the parameters of the studied tissue (knock-out mice vs. wild-type mice). The comparisons of results revealed high inter-rater reliability (Pearson R = 0.88).

### 2.6. Statistics

The mixed model was used for investigations. The parametric method was used throughout the analysis. Briefly, all calculations were performed using Statistica 13.3 (TIBCO Software Inc., Palo Alto, CA, USA). The differences between two independent groups (knock-out mice vs. wild-type mice) in statistical tests were calculated assuming that the statistical significance α = 0.05, and statistical power (1 − β) was equal to 0.95, and the allocation ratio was 1:1. Because the sample size of the knock-out mice group and the wild-type mice group was six in each subject, fifteen or ten sections were analysed, and the total number per group was ninety or sixty, respectively. The data from neurohistological research were averaged and examined for normal distribution by Shapiro–Wilk tests. The statistical graphs were produced by GraphPad Prism 6 software (GraphPad Software, La Jolla, CA, USA). The data are expressed as a box-and-whisker plots with the “box” depicting the median and 25th and 75th quartiles and “whiskers” showing the 5th and 95th percentile (*n* = 6). An independent-sample t-test was used to determine the differences between groups. The examined homogeneity of variance was used, and correction of Cochran’s Q test was then applied. The level of significance was set at *p* ≤ 0.05.

## 3. Results

The results of the present study demonstrate severe neuronal deficits in all main amygdala regions in ERβ knock-out mice,), accompanied by astroglia overexpression only in the medial, basomedial and cortical nuclei.Additionally, the density of CB+ neurons in the amygdala is significantly reduced in ERβ knock-out mice compared with matched controls, while the expressions of other markers of the GABAergic system such as PV, CR, SOM, GABA_A_α1 and VGAT are not affected by ERβ deficiency. 

### 3.1. Neuronal Deficits in the Amygdala of ERβ^−/−^ Mice

**Figure 1:**
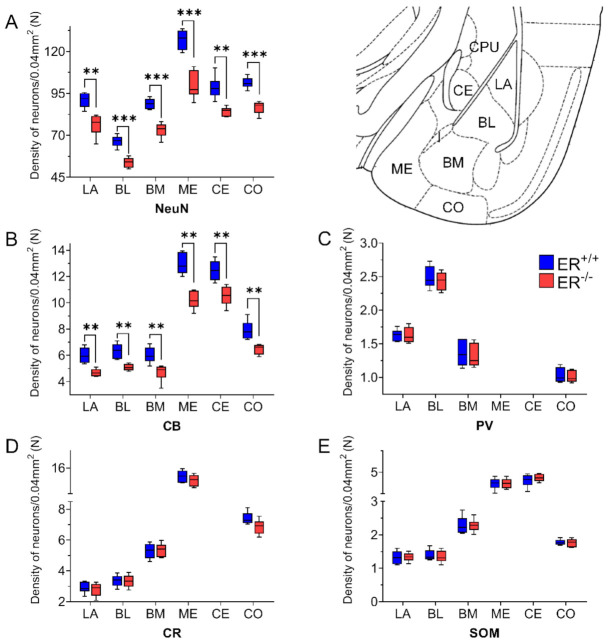
The density of neuron-specific nuclear protein (NeuN)-, calbindin (CB)-, parvalbumin (PV)-, calretinin (CR)- and somatostatin (SOM)-expressing neurons in the amygdala of wild-type (ERβ^+/+^) and ERβ and knock-out (ERβ^−/−^) mice. Note that the density of cells expressing NeuN (**A**) and CB (**B**) is significantly reduced in ERβ knock-out mice when compared with wild-type mice, while values for PV (**C**), CR (**D**) and SOM (**E**) did not differ in both ERβ^−/−^ and ERβ^+/+^ mice. Data are expressed as a box-and-whisker plots, with the “box” depicting the median and 25th and 75th quartiles and “whiskers” showing 5th and 95th percentile (*n* = 6). ** (*p* ≤ 0.01) and *** (*p* ≤ 0.001) indicate statistically significant differences between wild-type and ERβ knock-out mice. LA—lateral nucleus, BL—basolateral nucleus, BM—basomedial nucleus, ME—medial nucleus, CE—central nucleus, CO—cortical nucleus, I—intercalated nucleus, CPU—caudate-putamen complex.

**Figure 2:**
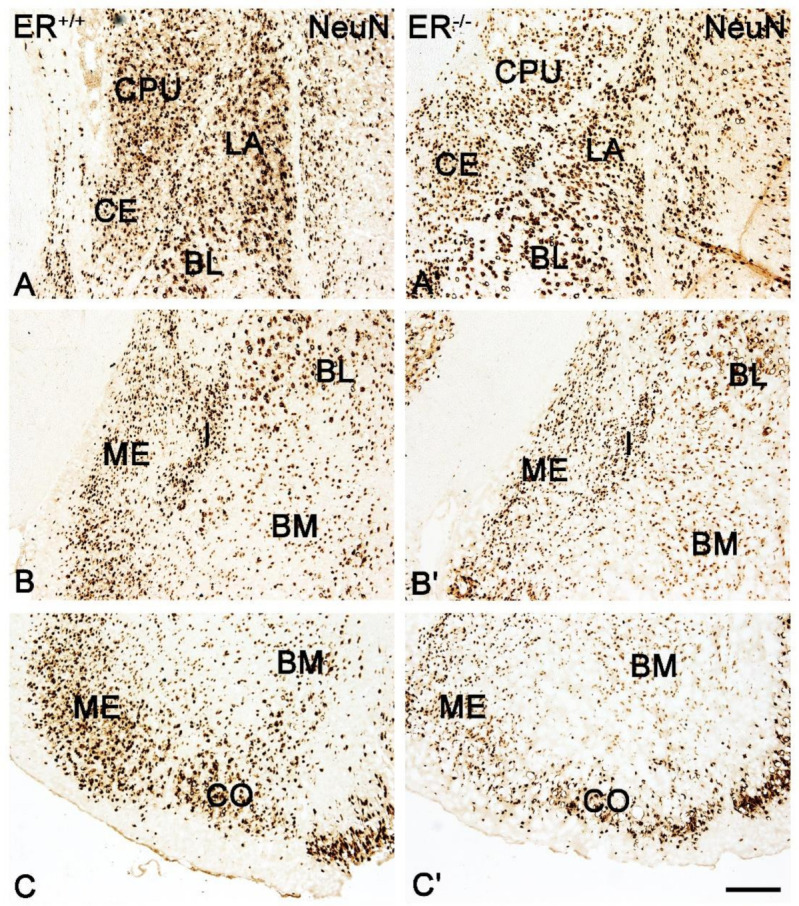
Brightfield photomicrographs illustrating the immunoreactivity patterns of neuron-specific nuclear protein (NeuN) in the amygdala of the wild-type (ERβ^+/+^, (**A**–**C**)) and ERβ knock-out (ERβ^−/−^, (**A′**,**C′**)) mice. (**A**,**A′**): The lateral (LA), basolateral (BL), central (CE) nuclei and caudate-putamen complex (CPU). (**B**,**B′**): The medial (ME), intercalated (I), basolateral (BL) and basomedial (BM) nuclei. (**C**,**C′**): The medial (ME), basomedial (BM) and cortical (CO) nuclei. Note the reduced density of NeuN+ neurons in the ERβ knock-out (**A′**–**C′**) subjects. Scale bar = 200 µm.

DAB staining with the use of an anti-NeuN antibody (pan-neuronal marker) revealed a significant neuronal deficit in all amygdala nuclei of ERβ^−/−^ mice when compared with ERβ^+/+^ (Figure 1A and Figure 2, Table 2). In the basolateral amygdala, the neuronal deficit was in a range of 16–19%. In the medial amygdala, the neuronal deficit was the most pronounced (~22%), while it was the smallest in the cortical and central amygdala (~14%).

### 3.2. Calcium-Binding Proteins in the Amygdala of ERβ^−/−^ Mice

**Figure 3:**
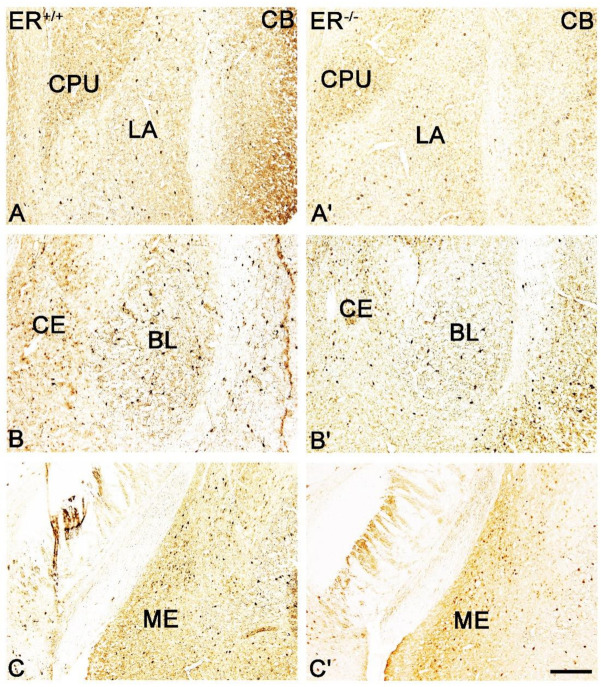
Brightfield photomicrographs highlighting the immunoreactivity patterns of calbindin (CB) in the amygdala of the wild-type (ERβ^+/+^, (**A**–**C**)) and ERβ knock-out (ERβ^−/−^, (**A′**–**C′**)) mice. (**A**,**A′**): The lateral (LA) nuclei and caudate-putamen complex (CPU). (**B**,**B′**): The basolateral (BL) and central (CE) nuclei. (**C**,**C′**): The medial (ME) nucleus. Note the reduced density of CB+ neurons in the ERβ knock-out (**A′**–**C′**) subjects. Scale bar = 200 µm.

**Figure 4:**
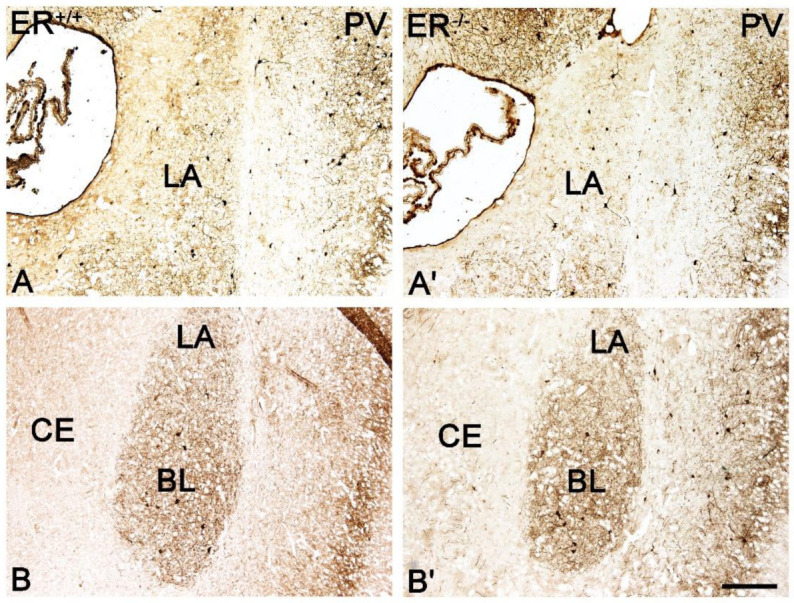
Brightfield photomicrographs highlighting the immunoreactivity patterns of parvalbumin (PV) in the amygdala of the wild-type (ERβ^+/+^, (**A**,**B**)) and ERβ knock-out (ERβ^−/−^, (**A′**,**B′**)) mice. (**A**,**A′**): The lateral (LA) nucleus. (**B**,**B′**): The lateral (LA), basolateral (BL) and central (CE) nuclei. Note similar density of PV+ neurons in the wild-type (**A**,**B**) and ERβ knock-out (**A′**,**B′**) subjects. Scale bar = 200 µm.

**Figure 5:**
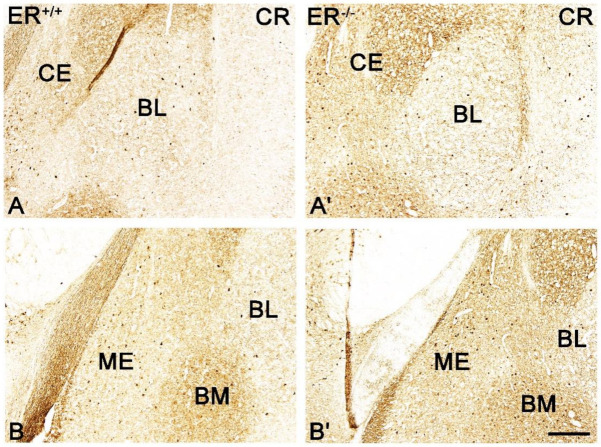
Brightfield photomicrographs highlighting the immunoreactivity patterns of calretinin (CR) in the amygdala of the wild-type (ERβ^+/+^, (**A**,**B**)) and ERβ knock-out (ERβ^−/−^, (**A′**,**B′**)) mice. (**A**,**A′**): The basolateral (BL) and central (CE) nuclei. (**B**,**B′**): The medial (ME), basolateral (BL) and basomedial (BM) nucleus. Note similar density of CR+ neurons in the wild-type (**A**,**B**) and ERβ knock-out (**A′**,**B′**) subjects. Scale bar = 200 µm.

**Figure 6:**
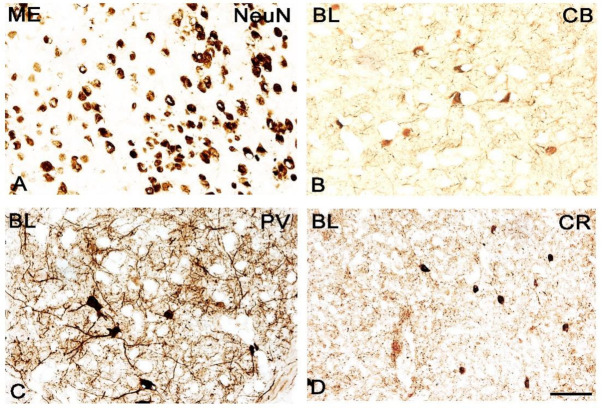
Brightfield photomicrographs illustrating the immunoreactivity of cellular structures of neuron-specific nuclear protein (NeuN, (**A**)), calbindin (CB, (**B**)), parvalbumin (PV, (**C**)) and calretinin (CR, (**D**)) of wild-type in the mice amygdala. (**A**): The medial (ME) and (**B**–**D**): The basolateral (BL). Scale bar = 50 µm.

Both the distribution and characteristics of CB+, PV+ and CR+ staining were comparable in ERβ^−/−^ and ERβ^+/+^ mice, and they closely matched earlier results from the adult rat brain [37]. The only exception was the CE in CR+ preparations which, similar to the guinea pig amygdala [55], had strongly stained neuropil but only a few immunopositive cells. As in other animals, CB+, PV+ and CR+ immunoreactivity in mice was mostly observed in perikarya and dendrites, but puncta were also present (Figure 3, Figure 4, Figure 5 and Figure 6). It is worth noting that in both ERβ^−/−^ and ERβ^+/+^ the distribution pattern for CB+, PV+ and CR+ in each nucleus gradually changed along a rostral to caudal gradient. However, this gradient was not observed among other markers studied. Despite the overall similarity in the immunoreactivity pattern in ERβ^−/−^ and wild-type mice, the density of CB+ neurons was substantially decreased in all amygdala regions of ERβ^−/−^ mice (Figure 1B and Figure 3, Table 2). In the basolateral and medial regions of the amygdala, the CB+ decrease was in the range of 19–22%. In the central and cortical regions of the amygdala, this decrease was also present, but it was less pronounced (15–18%). To the contrary, the density of PV+ and CR+ neurons was not affected due to ERβ deficiency (Figure 1C,D, Figure 4 and Figure 5, Table 2).

### 3.3. Somatostatin in the Amygdala of ERβ^−/−^ Mice

**Figure 7:**
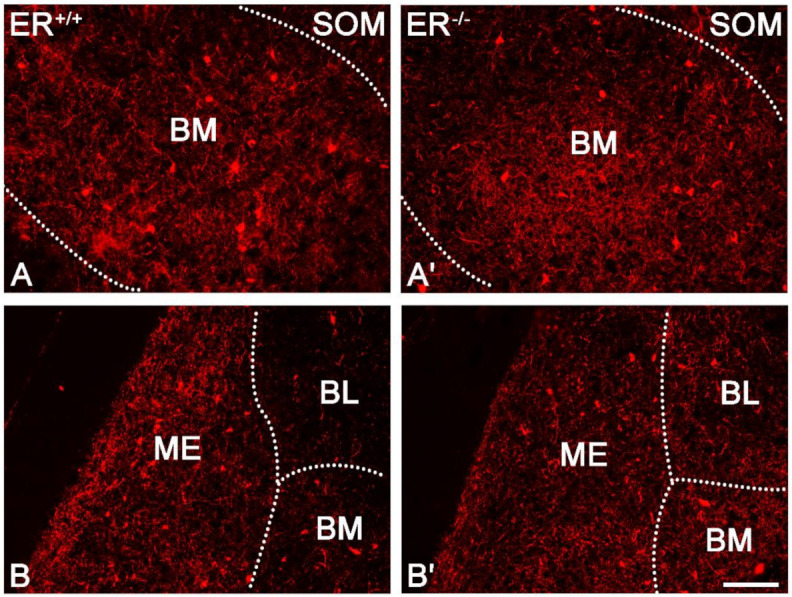
Representative colour photomicrographs illustrating the staining pattern of somatostatin (SOM) in the amygdala of wild-type (ERβ^+/+^, **(A**,**B**)) and ERβ knock-out (ERβ^−/−^, (**A′**,**B′**) mice**.** (**A**,**A′**): The basomedial (BM) nucleus. (**B**,**B′**): The medial (ME), basolateral (BL) and basomedial (BM) nucleus. Note similar density of SOM+ neurons in the wild-type (**A**,**B**) and ERβ knock-out (**A′**,**B′**) subjects. Scale bar = 100 µm.

The distribution and characteristics of somatostatin immunoreactivity were very similar in both ERβ^−/−^ and ERβ^+/+^ mice and did not differ from results reported previously within the adult mouse and rat brain [50]. Somatostatin staining was present in all amygdala nuclei, and it consisted mostly of somata and dendrites, although immunoreactive puncta were also present (Figure 7). Staining similarity was confirmed by a morphometric analysis since the density of SOM+ neurons was unchanged in all amygdala regions in ERβ^−/−^ mice when compared with ERβ^+/+^ mice (Figure 1E and Figure 7, Table 2).

### 3.4. GABA Type A Receptor with α1 Subunit in the Amygdala of ERβ^−/−^ Mice

**Figure 8:**
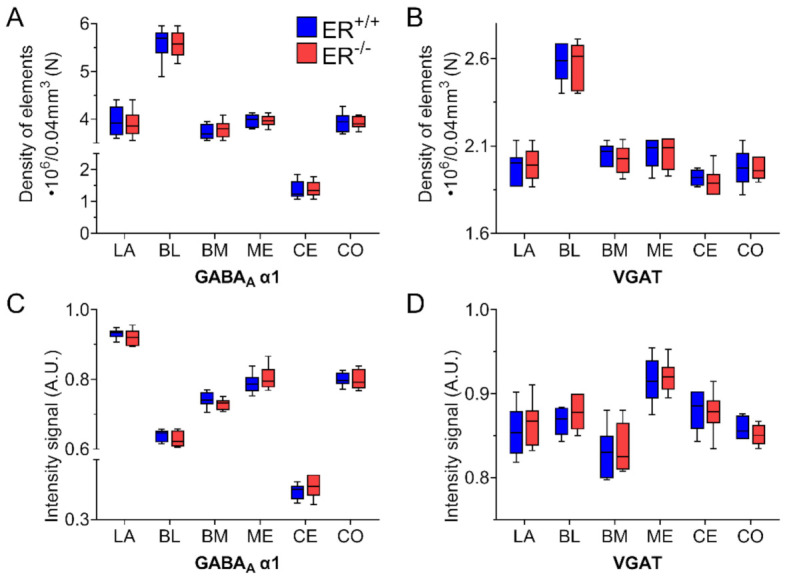
The volume density (elements) and signal intensity of GABA type A receptor with α1 subunit (GABA_A_α1) and vesicular GABA transporter (VGAT) in the amygdala of wild-type (ERβ^+/+^) and ERβ knock-out (ERβ^−/−^) mice. Note that the volume density (elements) of GABA_A_α1 (**A**) and VGAT (**B**) is similar in wild-type and ERβ knock-out mice. Note also that the signal intensity of GABA_A_α1 (**C**) and VGAT (**D**) is also similar in both ERβ^−/−^ and ERβ^+/+^ mice. Data are expressed as a box-and-whisker plots, with the “box” depicting the median and 25th and 75th quartiles, and “whiskers” showing 5th and 95th percentile (*n* = 6). LA—lateral nucleus, BL—basolateral nucleus, BM—basomedial nucleus, ME—medial nucleus, CE—central nucleus, CO—cortical nucleus.

**Figure 9:**
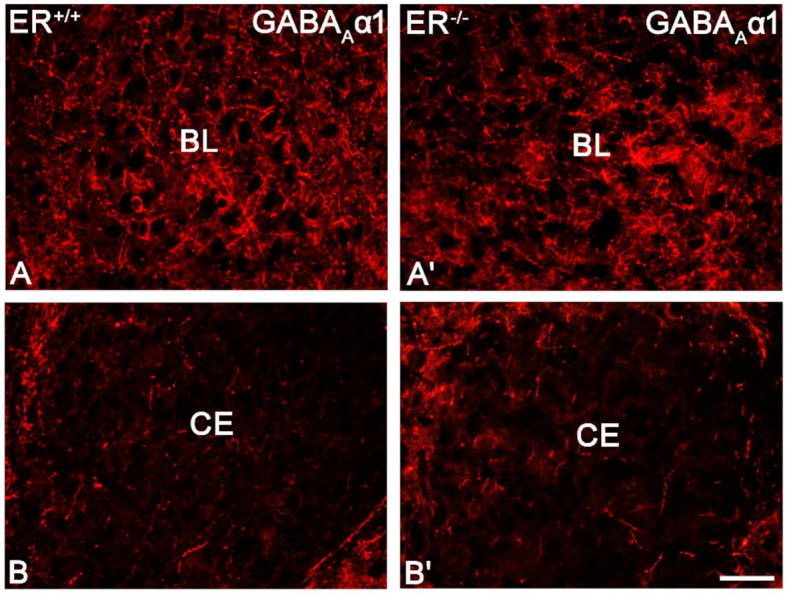
Representative colour photomicrographs illustrating the staining pattern of GABA type A receptor with α1 subunit (GABA_A_α1) in the amygdala of wild-type (ERβ^+/+^, (**A**,**B**)) and ERβ knock-out (ERβ^−/−^, (**A′**,**B′**)) mice. (**A**,**A′**): The basolateral (BL) nucleus. (**B**,**B′**): The central (CE) nucleus. Note similar density (elements) and signal intensity of GABA_A_α1 in the wild-type (**A**,**B**) and ERβ knock-out (**A′**,**B′**) subjects. IR elements—immunoreactive structures which include somata, fibres and puncta. Scale bar = 50 µm.

The distribution and characteristics of GABA_A_α1+ immunoreactivity were quite similar in both ERβ^−/−^ and ERβ^+/+^ mice, and they closely matched previous observations in the rodent amygdala [49,66,67,68]. GABA_A_α1+ staining was usually observed in dendrites, although positive somata and puncta were also frequently present (Figure 9). The immunoreactive elements in the amygdala were fairly uniformly distributed, except BL with the highest expression and CE with the lowest content (Figure 8A,C and Figure 9). Moreover, the cells were the most frequent in BL, whereas the most immunoreactive neuropil was present in the dorsal part of LA. As a large part of GABA_A_α1 immunoreactivity was actually present in dendrites, immunoreactive puncta densitometric analysis was performed. This analysis revealed a lack of any significant differences in the volume density and signal intensity of GABA_A_α1+ elements between ERβ^−/−^ and wild-type mice (Figure 8A,C, Table 2).

### 3.5. Vesicular GABA Transporter in the Amygdala of ERβ^−/−^ Mice

**Figure 10:**
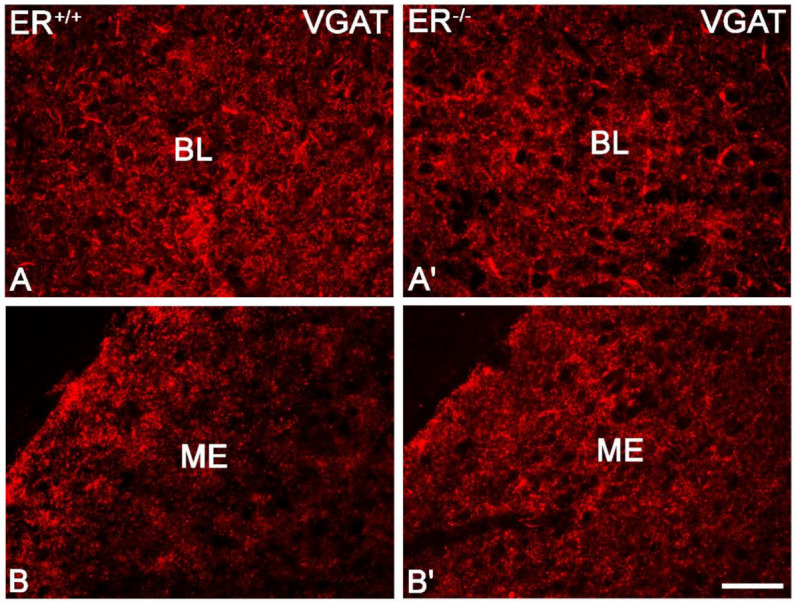
Representative colour photomicrographs illustrating the staining pattern of vesicular GABA transporter (VGAT) in the amygdala of wild-type (ERβ^+/+^, (**A**,**B**)) and ERβ knock-out (ERβ^−/−^, (**A′**,**B′**)) mice. (**A**,**A′**): The basolateral (BL) nucleus. (**B**,**B′**): The medial (ME) nucleus. Note similar density (elements) and signal intensity of VGAT in the wild-type (**A**,**B**) and ERβ knock-out (**A′**,**B′**) subjects. IR elements—immunoreactive structures which include somata, fibres and puncta. Scale bar = 50 µm.

The VGAT+ immunoreactivity pattern did not differ between ERβ^−/−^ and ERβ^+/+^ mice, and it generally consisted of immunoreactive puncta, although fibres were also present (Figure 10). The distribution of immunoreactive elements was rather homogenous and high in the whole amygdala except the BL with the highest content, although the signal intensity in the medial amygdala was slightly higher than in the basolateral region of the amygdala (Figure 8B,D and Figure 10). Densitometric comparisons did not show important differences in the volume density and signal intensity (Table 2).

### 3.6. Overexpression of Glial Fibrillary Acidic Protein in the Amygdala of ERβ^−/−^ Mice

**Figure 11:**
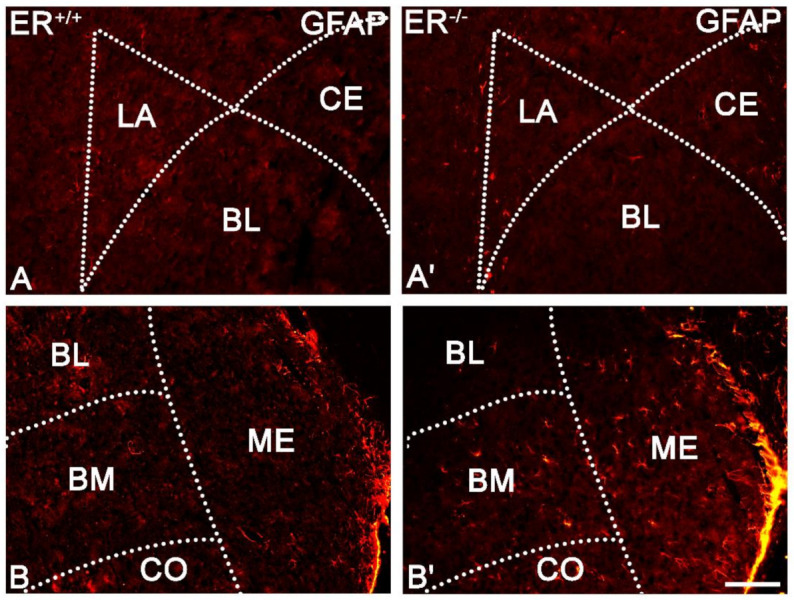
Representative colour photomicrographs illustrating the staining pattern of glial fibrillary acidic protein (GFAP) in the amygdala of wild-type (ERβ^+/+^, (**A**,**B**)) and ERβ knock-out (ERβ^−/−^, (**A′**,**B′**)) mice. (**A**,**A′**): The lateral (LA), basolateral (BL) and central (CE) nuclei. (**B**,**B′**): The basolateral (BL), basomedial (BM), cortical (CO) and medial (ME) nuclei. Note increased content of GFAP cells in the BM, CO and ME of ERβ knock-out subjects (**B′**). Note also the lack of such staining in the LA, BL and CE (**A′**). Scale bar = 100 µm.

GFAP-stained samples revealed significant overexpression of GFAP (astrocytes) in the ERβ knock-out mice compared with wild-type in selected amygdala nuclei, such as ME, BM and CO. However, the other regions such as LA, BL and CE were not affected in these mice (Figure 11).

## 4. Discussion

The results provide evidence of severe neuronal deficits in all main amygdala regions accompanied by astroglia overexpression in ME, BM and CO and show that the density of CB+ neurons, the largest subset of GABA+ neurons [37], in the amygdala of ERβ knock-out female mice is significantly reduced in comparison with ERβ^+/+^. In contrast, the density of PV+, CR+ and SOM+ neurons, as well as GABA_A_α1 and VGAT content, are unchanged in these mice. These results provide evidence that the lack of ERβ in mutant female mice affects the expression of CB in the GABAergic amygdala, causing a reduction in interneuron firing [69,70] and an eventual disinhibitory effect on pyramidal function [71]. This fear-promoting excitatory/inhibitory alteration may result in increased anxiety in these mice. The impact of neuronal deficits and astroglia overexpression on the amygdala functions is currently unknown.

The study demonstrated that in the amygdala of ERβ knock-out female mice, the density of CB+ neurons is significantly decreased relative to ERβ^+/+^ mice, while PV+ and CR+ cells remain unaffected. This coincides well with the fact that the CB reduction modifies fear, anxiety and social behaviours in mice, thus closely associating CB with emotional behaviours [72]. Importantly, since CB+, PV+ and CR+ neurons constitute the main subsets of GABA+ population which play fairly different roles in the amygdala intrinsic inhibitory system [42,43], any alterations in the activity of these populations may have a huge impact on this system and inhibitory/excitatory balance. However, it is unclear whether reduced CB expression in the amygdala of the ERβ knock-out mice reflects a real reduction in the CB+ cell numbers [73] or a decrease in protein expression [74]. The presence of CB deficit in these mice without any changes in PV and SOM markers, which are present in non-overlapping subpopulations comprising a large part of the CB+ population [50], strongly supports the latter hypothesis. Moreover, unchanged expressions of VGAT and GABA_A_α1 (present study), as well as GAD [24] which occurs in the majority of CB+ neurons, strengthens this hypothesis. However, severe neuronal deficits in all main amygdala regions (which contain many CB+ cells) and the existence of CB+ neurons (which do not co-express PV, SOM or GAD) [37,50] do not exclude the former hypothesis. Another important question is whether there is any compensation mechanism of the reduced CB expression. Schwaller [75] claims that neurons, once committed to expressing a particular calcium-binding protein (CaBP), are either incapable of turning on the expression of another EF-hand family member or that the distinct properties of any other CaBP would not suffice to restore “normal” Ca^2+^ homeostasis. On the other hand, Kreiner et al. [76] revealed that a consequence of loss of PV and CB is alterations in Cav2.1 channels. Modification of Cav2.1 properties through molecular switching of α12.1 splice variants and β subunits may represent a general mechanism for protecting neurons against aberrant Ca^2+^ signalling and pathological Ca^2+^ overloads [76]. Even when such compensation mechanisms exist, they may not be sufficient to protect neurons from abnormal Ca^2+^ signalling. At any rate, there are several mechanisms by which CB downregulation (even without an actual loss of CB+ neurons) may influence GABAergic inhibition. For example, a CB deficit would be expected to reduce interneuron firing [69] since downregulation of this protein renders the neuron in a less excitable state [70], probably due to the decreased capability to buffer transient intracellular Ca^2+^, which probably changes the duration of depolarisation and, perhaps more significantly, changes repolarisation events [71]. Persistent firing in such a situation may lead to a resting state in these neurons and will subsequently disinhibit the output of the pyramidal neurons [71]. One more mechanism concerning the CB reduction in ERβ knock-out mice is worth discussing. According to Schwaller [77], the absence of CB in neurons does not affect basal synaptic transmission but enhances facilitation and shortens delayed transmitter release [78,79]. Such a phenomenon is called “pseudofacilitation” because the main effect of CB leads to an amplitude decrease in inhibitory postsynaptic potential [77]. Interestingly, studies of CB knock-out mice revealed that animals of both sexes show less anxiety, indicating that CB lowering may have anxiolytic effects and may be a compensatory mechanism for maintaining GABAergic neurotransmission at a sufficient level [72].

The study also shows that the expressions of GABA_A_α1 receptor and VGAT were unchanged in ERβ knock-out female mice when tested by immunohistochemistry. Unchanged content of the expression of benzodiazepine-sensitive GABA_A_ receptors in these mice was also previously reported [24]. The expression of α1 subunit of GABA_A_ receptor also did not change in the amygdala of high anxiety-related behaviour (HAB) mice compared with matched control, although the content of other subunits (such as α5, β1, β2, γ1 and γ2) was altered in these mice [80]. Interestingly, GAD expression was unchanged in ERβ knock-out mice [24], while in HAB mice it increased [80], suggesting that the mechanism of increased anxiety is different in both these cases. The unchanged content of VGAT reported in the present study was also observed in the amygdala of helpless rats, one of the animal models of depression [81] and stressed aged mice [82].

ERβ knock-out mice have a significantly reduced number of neurons in all amygdala nuclei. Moreover, in the selected amygdala regions, such as ME, BM and CO, neuronal deficits are accompanied by astrocyte overexpression, which is not observed in other amygdala regions. The severity of neuronal deficits and astrocyte overexpression seems to coincide with ERβ distribution within the amygdala since ME, BM and CO are especially rich in ERβ in wild-type animals [50,83,84]. The present data are thus consistent with the studies of Wang et al. [27], who reported severe neuronal deficits and astroglia overexpression in ME of ERβ knock-out mice. A similar phenomenon was also reported in the amygdala as well as the hippocampus and prefrontal cortex of ovariectomised rats [51,85] under conditions similar to those in postmenopausal women. Interestingly, in these rats, GABA transporter type 3 (GAT3) was also overexpressed in the amygdala [51]. Since GAT3 is mainly synthetised in astrocytes [86] and GABA concentration in the synaptic gap is heavily controlled by glial cells [87], GAT3 overexpression may result in excessive uptake of GABA and reduced GABA availability in the synaptic cleft [86]. Thus, increased astroglia overexpression could impair overall GABA performance, resulting in the inhibitory/excitatory alteration and emotional disturbances observed in ovariectomised rats [51]. However, it seems that this is not the case in ERβ knock-out female mice since astrocyte overexpression is not present in LA, BL and CE, which are essential for fear processing [87]. Although the exact mechanism of neuronal deficits after ERβ knock-out is still unknown, there are several explanations. For example, ERβ is critically involved during brain development, and its disruption may promote the excessive death of neurons and/or incorrect neuronal migration in this period [88,89], which may partially explain neuronal deficits in quite young mice aged 8–10 weeks, as in the present results and Wang’s [27]. However, neuronal deficits increase with age [27], suggesting that there is also another mechanism after this period. Indeed, ERβ acts as a key element in neuron lifespan, and its disruption may lead to increased neuronal death [90]. The global disruption of ERβ may impair neuroprotective mechanisms and force neurons to die, which results in the neuronal deficits observed in the present study. It is noteworthy that neuronal deficits are also not restricted to the amygdala but are global since severe neuronal deficit in these mice was reported in the cortex, hypothalamus, ventral tegmental area, dorsal raphe nucleus and locus coeruleus; however, they were not present in the caudate-putamen, thalamus and hippocampus [27].

One more issue is worth discussing—i.e., what the clinical implications of the present results are. These results provide evidence that the expression of CB, a marker of the largest subset of GABA+ neurons [37], in the amygdala of ERβ knock-out female mice is significantly reduced as compared with ERβ^+/+^. Furthermore, in these mice there are severe cellular deficits in all regions of the amygdala, which increase with age [27]. Thus, in addition to ERβ [90], CB may be another potential therapeutic target that may help treat mood disorders in people with decreased oestrogen signalling, including menopausal women [51]. Interestingly, CB expression is decreased in ovariectomised rats, a state in which oestrogen levels are similar to those of menopausal women [51]. This view may be supported by the following facts. Alterations in CB content influence fear and anxiety in mice, evidencing that this protein is closely linked with emotional behaviours [72]. Furthermore, oestrogens have a direct effect on the transcription of the CB gene [91] via oestrogen-responsive elements in the CB promoter [91]. Thus, some effects of oestrogen therapy may be due to CB-promoting pathways. Finally, treatment with vitamin D in rats after ovariectomy increased CB levels, accompanied by a reduction in apoptosis, neuronal damage and depression-like behaviour [83]. Thus, the latter study indicates that clinical actions through CB-promoting pathways may also help prevent to some extent neurodegeneration in subjects with reduced ERβ signalling. As CB is involved in various psychiatric disorders [27,69,92,93,94,95], clinical actions through CB-promoting pathways may represent a new strategy for preventing and/or treating neurological diseases in women.

In conclusion, these data provide evidence that the density of CB+ neurons, the largest subset of GABAergic interneurons [37] in the amygdala of ERβ knock-out female mice, was significantly reduced as compared with matched controls, while the other markers of the GABAergic system remained unchanged. In addition, in these mice, there are severe neuronal deficits in all main amygdala regions, which are accompanied by astroglia overexpression in ME, BM and CO. Thus, it appears that the lack of ERβ in mutant female mice affects CB expression of GABAergic neurons, causing a reduction in interneuron firing [69] and, finally, a disinhibitory effect over pyramidal function [71]. This fear-promoting excitatory/inhibitory alteration may lead to the increased anxiety observed in these mice. The impact of neuronal deficits and astroglia overexpression on the amygdala functions is still unknown.

## Figures and Tables

**Table 1:** Specification of reagents.

Antigen	Code	Clonality	Host Species	Dilution	Supplier	Location
Primary antibodies						
NeuN	ABN78	polyclonal	Rabbit	1:1000	Millipore	Temecula, CA, USA
CB	CB-38	polyclonal	Rabbit	1:4000	SWANT	Bellinzona, Switzerland
PV	PV27	polyclonal	Rabbit	1:1000	SWANT	Bellinzona, Switzerland
CR	7697	polyclonal	Rabbit	1:1000	SWANT	Bellinzona, Switzerland
SOM	MAB354	monoclonal	Rat	1:1000	Millipore	Temecula, CA, USA
GABA_A_ α1	AB33299	polyclonal	Rabbit	1:2000	Abcam	Cambridge, UK
VGAT	AB5062P	polyclonal	Rabbit	1:3000	Millipore	Temecula, CA, USA
GFAP	G9269	polyclonal	Rabbit	1:200	Millipore	Temecula, CA, USA
Secondary antibodies						
ImmPRESS HRP Universal Antibody (anti-rabbit Ig, Peroxidase)				1:1	Vector Laboratories	Burlingame, CA, USA
ALEXA Fluor 555	A-31572	polyclonal	Donkey anti-rabbit	1:1000	Thermo Fisher	Rockford, IL, USA
CY3	712-165-153	polyclonal	Donkey anti-rat	1:1000	Jackson ImmunoResearch Laboratories	West Grove, PA, USA
Other reagents						
3,3-diaminobenzidine substrate chromogen				3%	Dako Cytomation	Glostrup, Denmark

**Table 2:** Statistical analysis of studied markers between ERβ^+/+^ and ERβ^−/−^ mice in the amygdala.

	LA		BL		BM		ME		CE		CO	
		*p*		*p*		*p*		*p*		*p*		*p*
NeuN	t_8.88_ = 4.77	0.001057	t_9.70_ = 6.88	0.000051	t_9.02_ = 7.25	0.000048	t_8.98_ = 6.74	0.000085	t_9.78_ = 7.05	0.000039	t_6.66_ = 4.86	0.002112
CB	t_7.27_ = 4.74	0.001913	t_7.27_ = 4.74	0.002045	t_9.55_ = 4.16	0.004259	t_9.58_ = 6.33	0.001030	t_9.99_ = 4.46	0.001229	t_7.80_ = 4.40	0.002435
PV	t_10_ = 0.054	0.9581	t_10_ = 0.62	0.5482	t_10_ = 0.48	0.6642	−	−	−	−	t_10_ = 0.46	0.6581
CR	t_10_ = 0.53	0.6113	t_10_ = 0.16	0.8787	t_10_ = −0.24	0.8119	t_10_ = 1.24	0.2426	−	−	t_10_ = 2.13	0.0594
SOM	t_10_ = −0.22	0.8274	t_10_ = 0.08	0.9344	t_10_ = −0.26	0.8036	t_10_ = 0.17	0.8662	t_10_ = 0.63	0.5441	t_10_ = 0.16	0.8732
GABA_A_α1	t_10_ = 0.35	0.7337	t_10_ = 0.11	0.9178	t_10_ = −0.62	0.5478	t_10_ = 0.08	0.9357	t_10_ = −0.19	0.8513	t_10_ = 0.11	0.9106
Intensity of GABA_A_α1	t_10_ = 0.94	0.3674	t_10_ = 0.97	0.3536	t_10_ = 1.24	0.2445	t_10_ = −0.82	0.4334	t_10_ = −0.85	0.4133	t_10_ = −0.08	0.9379
VGAT	t_10_ = −0.22	0.8274	t_10_ = 0.08	0.9344	t_10_ = −0.26	0.8036	t_10_ = 0.17	0.8662	t_10_ = 0.63	*p* = 0.5441	t_10_ = 0.16	0.8732
Intensity of GABA_A_α1	t_10_ = −0.57	0.5829	t_10_ = −1.00	0.3432	t_10_ = −0.29	0.7796	t_10_ = −0.31	0.7607	t_10_ = 0.19	*p* = 0.8495	t_10_ = 1.07	0.3091

Lack of immunoreactive structures was marked as a dash.

## Data Availability

The data that support the findings of this study are available from the corresponding author upon reasonable request.
